# Conventional Synthetic Disease-Modifying Anti-rheumatic Drugs for Psoriatic Arthritis: Findings and Implications From a Patient Centered Longitudinal Study in Brazil

**DOI:** 10.3389/fphar.2022.878972

**Published:** 2022-04-26

**Authors:** Ronaldo José Faria, Francisca Janiclecia Rezende Cordeiro, Jéssica Barreto Ribeiro dos Santos, Juliana Alvares-Teodoro, Augusto Afonso Guerra Júnior, Francisco de Assis Acurcio, Michael Ruberson Ribeiro da Silva

**Affiliations:** ^1^ Pharmaceutical Services Graduate Program, Federal University of Espírito Santo, Alegre, Brazil; ^2^ Health Assessment, Technology, and Economy Group, Federal University of Espírito Santo, Alegre, Brazil; ^3^ Department of Social Pharmacy, Federal University of Minas Gerais, Belo Horizonte, Brazil

**Keywords:** psoriatic arthritis, pharmacoepidemiology, antirheumatic agents, medication adherence, drug costs

## Abstract

**Background:** Conventional synthetic disease-modifying antirheumatic drugs are the first-line treatment to inhibit the progression of psoriatic arthritis. Despite their widespread clinical use, few studies have been conducted to compare these drugs for psoriatic arthritis.

**Methods:** a longitudinal study was carried out based on a centered patient national database in Brazil. Market share of drugs, medication persistence, drug costs, and cost per response were evaluated.

**Results:** a total of 1,999 individuals with psoriatic arthritis were included. Methotrexate was the most used drug (44.4%), followed by leflunomide (40.6%), ciclosporin (8.2%), and sulfasalazine (6.8%). Methotrexate and leflunomide had a greater market share than ciclosporin and sulfasalazine over years. Medication persistence was higher for leflunomide (58.9 and 28.2%), followed by methotrexate (51.6 and 25.4%) at six and 12 months, respectively. Leflunomide was deemed the most expensive drug, with an average annual cost of $317.25, followed by sulfasalazine ($106.47), ciclosporin ($97.64), and methotrexate ($40.23). Methotrexate was the drug being the lowest cost per response.

**Conclusion:** Methotrexate had the best cost per response ratio, owing to its lower cost and a slightly lower proportion of persistent patients when compared to leflunomide. Leflunomide had a slightly higher medication persistence than methotrexate, but it was the most expensive drug.

## Introduction

Psoriatic arthritis (PsA) is a chronic inflammatory musculoskeletal disease with a wide range of symptoms. The four domains of musculoskeletal involvement in PsA are peripheral arthritis, dactylitis, enthesitis, and axial arthritis. Other non-musculoskeletal symptoms, such as uveitis, inflammatory bowel disease, nail psoriasis, and elevated acute phase reactants, help to diagnose PsA. Early diagnosis and treatment are difficult due to the non-specific and often subtle symptoms (Rida and Chandran, 2020).

The treatment of PsA has changed substantially over the past 10 years (Ogdie et al., 2020). Clinical practice guidelines have been created to assist clinicians in quickly integrating new therapeutic management knowledge into their practice. Treatment for PsA includes conventional synthetic disease-modifying antirheumatic drugs (csDMARDs), biologic therapies such as TNF inhibitors (TNFi), IL-17 inhibitors (IL-17i), IL-12/23 inhibitor (IL-12/23i), and new targeted oral agents including a phosphodiesterase-4 inhibitor and Janus kinase (JAK)/signal transducer and activator of transcription (STAT) inhibitors (Coates and Helliwell, 2015; Gossec et al., 2015; Singh et al., 2018; Ogdie et al., 2020).

Synthetic drugs have been used to treat psoriatic arthritis since 1964. However, their use is largely derived from their utilization for rheumatoid arthritis (RA) and there is little evidence of clinical efficacy, usually restricted to peripheral outcomes for the short-term, without consistent long-term efficacy data (Coates and Helliwell, 2015). Methotrexate is known to be safe and effective in the treatment of RA and psoriasis, and it has been used to treat PsA despite scarce evidence from randomized controlled trials to support it. Some observational studies have supported the use of MTX, and current treatment recommendations approve its use as a first-line agent for the management of psoriatic arthritis with predominant peripheral arthritis (Elmanoum and Chandran, 2018; Coates et al., 2020). Furthermore, other csDMARD have also shown limited evidence of efficacy for the treatment of PsA (Kang and Kavanaugh, 2015).

Depending on the main impairment presented by the patient, the treatment takes different approaches. The EULAR and GRAPPA guidelines recommend starting with a csDMARD in most patients with treatment-naive predominantly peripheral arthritis. In addition, the GRAPPA guideline suggests that a biologic may be selected first if the situation warrants more aggressive therapy. Unless there are contraindications, EULAR recommends starting with methotrexate (MTX) as the first csDMARD. This recommendation was based on the efficacy of MTX in RA, similar medication persistence among patients with PsA and RA treated with MTX, data from the Tight Control in Psoriatic Arthritis trial, and expert opinion. The EULAR recommendations recognized the lack of data available at the time to support the use of MTX in clinical trials (Gossec et al., 2015; Coates et al., 2016; Singh et al., 2018; Ogdie et al., 2020).

In this sense, this study aimed to assess market share, medication persistence, drug costs, and cost per response in the treatment of PsA with csDMARD. Thus, a performance evaluation of the drugs available in Brazilian public health system was carried out to identify those with better performance and generate real world evidence for the treatment PsA.

## Methods

### National Health Database

A National Health Database centered on the individual was created to conduct clinical, epidemiological, and economic studies using real-world evidence. This National Database incorporated health data from all 26 Brazilian states and the Federal District of individuals that used the Public National Health System. The data include records of inpatient care, outpatient care, and deaths from January 2000 to December 2015 (Guerra Junior et al., 2018). Psoriatic arthritis treatment was officially introduced in Brazil in 2010. As a result, the study’s follow-up period lasted from 2010 to 2015. The data did not include information about the Brazilian private market, such as direct disbursements by individuals or health insurance coverage.

### Patients and Market Share

Patients diagnosed with PsA according to Classification Criteria for Psoriatic Arthritis (CASPAR), with codes M07.0 and M07.3 from the International Classification of Diseases 10th version (ICD-10), who utilized cyclosporin, leflunomide, methotrexate, and sulfasalazine as first-line treatment in monotherapy were included. Patients using biological drugs concomitantly, with other osteoarticular inflammatory diseases or who had an absolute contraindication to the use of csDMARD were not eligible.

The first date of drug dispensation for the treatment of PsA was used to determine the date of entry into the follow-up. All patients were followed up on until their deaths or the end of the follow-up period.

Market share was assessed annually by identifying the number of patients being treated per drug in use in the public sector.

### Medication Persistence

Medication persistence has been used as a proxy for effectiveness and safety of using antirheumatic agents (Luttropp et al., 2019; Ribeiro da Silva et al., 2019; Souza et al., 2021).

The absence of medication dispensation after 90 days from the last date of dispensation, a period corresponding to treatment renewal by SUS, was considered treatment discontinuation. The time between the first and last dispensation, plus a 30-days grace period (medication possession), was used to calculate the time until discontinuation. The proportion of people who remained on treatment was assessed after 6 and 12 months of follow-up for each drug. In addition, medication persistence in 18 and 24 months was presented.

Sensitivity analysis through propensity score weighting was used to control confounders at baseline and adjust the results to these. That is why inverse-probability weights were used to estimate the average treatment effect (ATE) on discontinuation time among drugs (Austin and Stuart, 2015).

Variables with statistically significant differences at baseline at a 5% significance level were included as balancing variables in the propensity score weighting.

### Costs and Cost per Response

Cost analysis was developed from the perspective of the Brazilian Public Health System. The annual average direct costs with csDMARD were estimated using the macro-costing approach (top-down). The cost per response was calculated by dividing the costs by the response rate in 1 year of treatment.

The World Bank’s conversion factor “purchasing power parity” (PPP) was used to adjust the monetary values. PPP rates are annual and provide a standard measurement by which countries’ expenditure levels can be compared (World Bank, 2020).

The cost per response was calculated by dividing the annual drug cost by the observed medication persistence at 12-months follow-up period.

### Statistical Analysis

Frequency distribution tables were elaborated for the categorical variables, and average with standard deviation (SD) or a confidence interval of 95% (CI95%) for the continuous variables. Kaplan-Meier curves were estimated to verify the time up to treatment discontinuation, that is, the loss of medication persistence. The log-rank test was used to verify if there were any differences among the groups for medication persistence.

Regression by the model of Cox proportional risks was used to verify the predictors of treatment discontinuation. Independent variables included in the model were age, sex, region of residence, csDMARD used, fragility index, and Charlson comorbidity index. A significance level of 20% was used for the bivariate analyses, and 5% was adopted for the multivariable analysis.

The Charlson comorbidity index, adapted from Quan et al. (2005), predicts mortality through the ponderation of patient comorbidities and it was used to measure the burden of the disease. The index score was calculated using data from outpatient and hospital medical services 3 years before entry into the cohort according 19 specified conditions. An index score of 0 indicates no comorbid conditions, while higher scores indicate a greater level of comorbidity (Quan et al., 2005). Days of hospitalizations for any cause were accounted for 2 years before the entry into the cohort, as a patient general frailty index (Neovius et al., 2013). The Charlson and frailty index were used as baseline indicators of general health in the study, which are related to occurrence comorbidities and hospitalizations, respectively.

Costs were compared through Analysis of Variance (ANOVA) with posthoc Bonferroni analysis. The analyses were developed using the software Stata^®^ (Statistics/Data Analysis) version 16.1.

## Results

### Sociodemographic and Clinical Characteristics of Patients

The study included 1,999 individuals with PsA on first-line treatment with csDMARD. The mean age of the patients was 51.11 years (12.77), with a predominance of females (60.1%). Most individuals resided in the Southeast and South regions, mainly in the states of São Paulo, Paraná, Rio Grande do Sul, Minas Gerais, and Santa Catarina. In contrast, the Northern region of the country represents only 0.8% of the study population. During follow-up, it was observed that 14.4% of the individuals experienced hospital admission. About 29% of patients had at least one out of 19 conditions specified by the Charlson Index (Table 1).

**TABLE 1:** Baseline characteristics of PsA patients who used csDMARD.

Variables	csDMARD (n = 1.999)	Ciclosporin (n = 164)	Leflunomide (n = 812)	Methotrexate (n = 887)	Sulfasalazine (n = 136)	*p*-value	Obs
**Female n (%)**	1202 (60.1)	75 (45.7)	533 (65.6)	515 (58.1)	79 (58.1)	<0.001	^a^
**Male n (%)**	797 (39.9)	89 (54.3)	279 (34.4)	372 (41.9)	57 (41.9)		
**Age in years mean (SD)**	51.11 (12.77)	46.68 (13.68)	52.05 (12.22)	51.26 (12.73)	49.89 (14.03)	<0.001	^b^
**Region or residence n (%)**	—	—	—	—	—	<0.001	^c^
Southeast	1022 (51.1)	95 (57.9)	431 (53.1)	421 (47.5)	75 (55.1)	—	—
South	754 (37.7)	35 (21.3)	286 (35.2)	394 (44.4)	39 (28.7)	—	—
Northeast	138 (6.9)	18 (11.0)	59 (7.3)	51 (5.7)	10 (7.4)	—	—
Central west	69 (3.4)	15 (9.1)	29 (3.6)	19 (2.1)	6 (4.4)	—	—
North	16 (0.8)	1 (0.6)	7 (0.9)	2 (0.2)	6 (4.4)	—	—
**State of residence n (%)**	—	—	—	—	—	<0.001	^c^
São Paulo	686 (34.2)	75 (45.7)	248 (30.5)	298 (33.6)	65 (47.8)	—	—
Paraná	256 (12.8)	20 (12.2)	44 (5.4)	178 (20.1)	14 (10.3)	—	—
Rio Grande do Sul	299 (15.0)	2 (1.2)	156 (19.2)	126 (14.2)	15 (11.0)	—	—
Minas Gerais	195 (9.8)	4 (2.4)	103 (12.7)	83 (9.4)	5 (3.7)	—	—
Santa Catarina	199 (10.0)	13 (7.9)	86 (10.6)	90 (10.1)	10 (7.4)	—	—
Outros	364 (18.2)	50 (30.6)	175 (21.6)	112 (12.6)	27 (19.8)	—	—
**Frailty index n (%)**	288 (14.4)	22 (13.4)	93 (11.5)	155 (17.5)	18 (13.2)	0.567	^d^
**Frailty index mean (SD)**	1.36 (5.77)	1.47 (5.68)	1.24 (6.42)	1.53 (5.41)	0.87 (3.76)	0.324	^d^
**Charlson index n (%)**	576 (28.8)	40 (24.4)	267 (32.9)	220 (25.8)	49 (36.0)	<0.001	^e^
**Charlson index mean (*SD***)	0.40 (0.87)	0.38 (0.93)	0.43 (0.80)	0.36 (0.82)	0.60 (1.36)	0.094	^f^
**Gini index mean (*SD***)	0.52 (0.07)	0.538 (0.077)	0.519 (0.071)	0.511 (0.071)	0.515 (0.070)	<0.001	^g^

CsDMARD, conventional synthetic DMARD., Obs, observation.

a significant for all comparisons, except for methotrexate versus sulfasalazine.

b significant only for ciclosporin versus methotrexate and ciclosporin versus leflunomide.

c significant for all comparisons.

d no significance for all comparisons.

e significant for all comparisons, except for methotrexate versus ciclosporin and ciclosporin versus sulfasalazine.

f significant only for sulfasalazine versus methotrexate and sulfasalazine versus ciclosporin.

g significant for all comparisons, except for sulfasalazine versus methotrexate and sulfasalazine versus leflunomide.

Methotrexate was the most used drug by patients (44.4% n = 887), followed by leflunomide (40.6%), ciclosporin (8.2%), and sulfasalazine (6.8%), as shown in Table 1.

### Market Share of csDMARD

**FIGURE 1:**
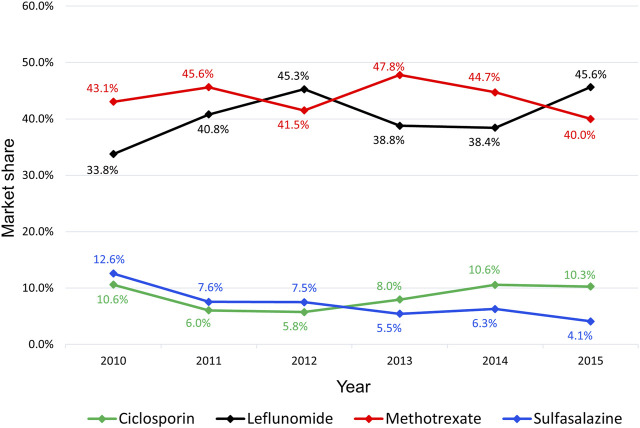
Market share of csDMARD for psoriatic arthritis from 2010 to 2015.

Methotrexate had a market share ranging from 41 to 48%, occasionally alternating the leading with leflunomide, which had a market share ranging from 34 to 46%. The market share of sulfasalazine and ciclosporin was lower than methotrexate and leflunomide. Sulfasalazine’s market share has decreased over time, reaching 4% in 2015, whereas ciclosporin has maintained a market share of around 10% over time (Figure 1).

### Medication Persistence

At 6-months follow-up, 53.4% of patients persisted in treatment with a mean time until to treatment discontinuation of 153.74 days (151.96–155.52). Patients treated with leflunomide presented highest medication persistence (58.9%; n = 478), followed by those treated with methotrexate (51.6%; n = 458). Patients taking sulfasalazine (44.8% n = 61) and ciclosporin (42.7%; n = 70) had a lower medication persistence (*p* < 0.001).

At the end of the first year of follow-up, patients using leflunomide remained with the slightly higher medication persistence (28.2% n = 229) than patients using methotrexate (25.4% = 458). Similar to the 6-month follow-up analysis, patients who used ciclosporin and sulfasalazine for 12 months maintained a lower medication persistence. Patients using leflunomide presented higher medication persistence than ones using other csDMARD (*p* < 0.05) (Table 2).

**TABLE 2:** Medication persistence at 6 and 12 months of follow-up.

Drug	Medication Persistence n (%)	Time until Discontinuation Mean (CI 95%)	Medication Persistence n (%)	Time until Discontinuation Mean (CI 95%)
	6 months		12 months	
Leflunomide (n = 812)	478 (58.9)	159.89 (157.41–162.38)	229 (28.2)	237.68 (230.36–245.00)
Methotrexate (n = 887)	458 (51.6)	150.90 (148.03–153.76)	225 (25.4)	219.06 (211.67–226.45)
Sulfasalazine (n = 136)	61 (44.8)	144.09 (136.83–151.14)	32 (19.5)	199.34 (183.24–215.45)
Ciclosporin (n = 164)	70 (42.7)	146.61 (140.49–152.73)	24 (17.6)	195.84 (177.97–213.71)
Total (n = 1.999)	1,067 (53.4)	153.74 (151.96–155.52)	510 (25.5)	223.43 (218.62–228.23)
*p*-value	<0.001^a^	<0.001^b^	0.014^c^	<0.001^b^

a significant for leflunomide versus methotrexate, leflunomide versus sulfasalazine, leflunomide versus ciclosporin, and methotrexate versus ciclosporin. No differences for other comparisons.

b significant for leflunomide versus methotrexate, leflunomide versus sulfasalazine, and leflunomide versus ciclosporin. No differences for other comparisons.

c significant for leflunomide versus sulfasalazine and leflunomide versus ciclosporin. No differences for other comparisons.

**FIGURE 2:**
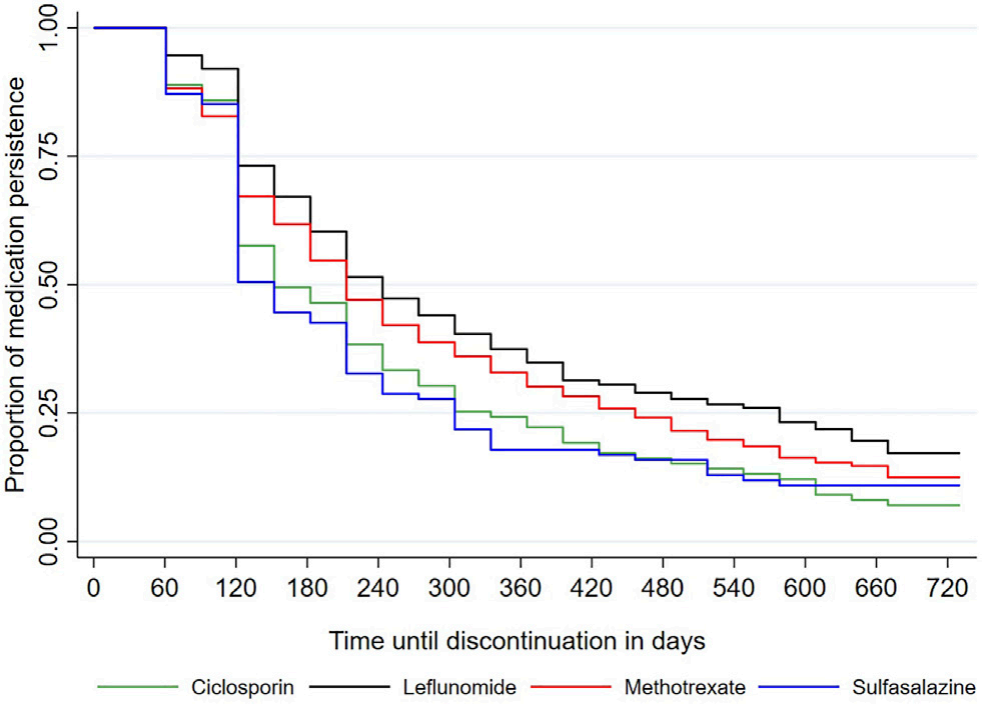
Medication persistence of csDMARD for psoriatic arthritis.

In addition, patients taking leflunomide were more persistent in treatment at 18 (18.5%) and 24 (12.2%) months. Methotrexate comes next with 13.1% (18 months) and 8.8% (24 months) of medication persistence. Moreover, patients treated with sulfasalazine and ciclosporin had a higher discontinuation rate, with only 8.8% (18 months) and 8.1% (24 months) of the patients initially treated with sulfasalazine and 7.9% (18 months) and 4.3% (24 months) of those treated with ciclosporin persisting with therapy (log-rank < 0,05) (Figure 2).

Sensitivity analysis confirmed the original findings, with patients taking leflunomide maintaining higher medication persistence than patients taking the other drugs after covariates balance at baseline (Table 3).

**TABLE 3:** Average treatment effect after propensity score weighting: pairwise analyses.

Pairwise Comparison	6 months				12 months		
	ATE	CI 95%	*p*-value		ATE	CI 95%	*p*-value
MTX vs. LEF	−9.91	−13.68; −6.13	<0.001	MTX vs. LEF	−21.76	−32.01; −11.51	<0.001
CCP vs. LEF	−10.70	−11.21; −4.65	0.001	CCP vs. LEF	−36.85	−55.61; −18.09	<0.001
SSZ vs. LEF	−15.53	−22.90; −8.16	<0.001	SSZ vs. LEF	−40.99	−60.81; −21.17	<0.001
CCP vs. MTX	−1.25	−7.89; 5.39	0.712	CCP vs. MTX	−14.71	−31.21; 3.79	0.119
SSZ vs. MTX	−5.91	−13.45; 1.62	0.124	SSZ vs. MTX	−19.53	−39.32; 0.26	0.053
SSZ vs. CCP	−3.89	−13.24; 5.45	0.414	SSZ vs. CCP	−4.48	−29.65; 20.69	0.727

CCP: ciclosporin; LEF: leflunomide; MTX: methotrexate; SSZ: sulfasalazine. ATE: average treatment effect.

At 12-months of follow-up, approximately 66% of non-persistent patients discontinued the treatment, while 34% switched or added a new medication to the treatment. Sulfasalazine and cyclosporine had a higher proportion of treatment discontinuations than leflunomide and methotrexate. Among the patients who switched or added drugs to the treatment, most started using biological drugs, especially adalimumab (Table 4).

**TABLE 4:** Withdrawal and switch treatments at 12 months.

Ciclosporin n (%)		Leflunomide n (%)		Methotrexate n (%)		Sulfasalazine n (%)	
Withdraw	Switch	Withdraw	Switch	Withdraw	Switch	Withdraw	Switch
90 (68.2)	42 (31.8)	348 (59.7)	235 (40.3)	406 (61.3)	256 (38.7)	74 (66.1)	38 (33.9)
**Switch - 42 (100)**		**Switch - 235 (100)**		**Switch - 256 (100)**		**Switch - 38 (100)**	
**22 (55.4) = ADA**		**100 (42.5) = ADA**		**91 (35.5) = ADA**		**10 (26.3) = ADA**	
**9 (21.4) = ETA**		**62 (26.4) = ETA**		69 (27.0) = LEF		9 (23.7) = MTX	
56 (14.3) = LEF		53 (22.6) = MTX		**48 (18.8) = ETA**		9 (23.7) = LEF	
**3 (7.4) = IFX**		**16 (6.8) = IFX**		**26 (10.2) = IFX**		**8 (21.1) = ETA**	
2 (7.4) = MTX or SSZ		12 (5.1) CCP or SSZ		12 (4.7) = SSZ		**3 (7.9) = IFX**	

ADA: adalimumab; CCP: ciclosporin; ETA: etanercept; IFX: infliximab; LEF: leflunomide; MTX: methotrexate; SSZ: sulfasalazine. Bold: biologic DMARD.

### Predictors of Non-persistence in the Use of csDMARD

The predictors of non-persistence to treatment were younger patients, living in the northern and northeastern regions of the country, and who were not on leflunomide. Thus, it was possible to identify that the risk of treatment discontinuation decreases with increasing age (Hazard ratio [HR] = 0.995, 95% confidence interval [95% CI] 0.991–0.991). The risk of treatment discontinuation of the northeastern and northern regions was 75% higher than south, southeast, and central-west of Brazil (HR = 1.750, 95% CI 1,471–2,084). Finally, patients using methotrexate, sulfasalazine, and ciclosporin also have a higher risk of discontinuing treatment. Patients taking sulfasalazine had a hazard risk of 1.39 for discontinuation (39% higher), followed by ciclosporin with 1.30 (30% higher), and methotrexate with 1.16 (16% higher) compared to leflunomide (Table 5). Socioeconomic inequality (measured by GINI), comorbidity index, and frailty index were not identified as predictors of treatment discontinuation.

**TABLE 5:** Predictors of treatment discontinuation at 12 months of follow-up.

Variables	Crude HR (CI 95%)	*p*-value	Adjusted HR (CI 95%)	*p*-value
**Sex**				
Female	1	—	—	—
Male	0.965 (0.869–1.170)	0.500	—	—
**Age**	0.994 (0.990–0.998)	0.002	0.995 (0.991–0.999)	0.010
**Region**				
South/Southeast/Central west	1	—	1	—
Northeast/North	1.779 (1.495–2.116)	<0.001	1.750 (1.471–2.084)	<0.001
**CsDMARD**				
Leflunomide	1	—	1	—
Methotrexate	1.150 (1.029–1.744)	0.014	1.160 (1.038–1.297)	0.009
Sulfasalazine	1.424 (1.163–1.744)	0.001	1.387 (1.133–1.699)	0.002
Ciclosporin	1.371 (1.135–1.657)	0.001	1.297 (1.071–1.570)	0.008
**GINI**	3.522 (1.707–7.271)	0.001	—	—
**Charlson Index**	1.055 (1.002–1.110)	0.040	—	—
**Frailty Index**	1.006 (0.998–1.119)	0.119	—	—

HR, hazard ratio; CI95% = Confidence interval 95%; csDMARD, conventional synthetic DMARD.

### Drug Costs and Cost per Response

The mean annual cost per patient was $105.32 (171.34) at 12 months of follow-up, and a statistically significant difference was observed in the spending among drugs (*p* < 0.001), except for ciclosporin versus sulfasalazine. Leflunomide was considered the drug with the highest cost, with an average of $317.25, followed by sulfasalazine ($106.47), ciclosporin ($97.64), being methotrexate the lowest cost drug ($40.23) (Table 6).

**TABLE 6:** Drug costs and cost per response of csDMARD.

csDMARD	Annual cost		Response Rate	Cost per Response		Rank
	BRL	PPP dolar		BRL	PPP dolar	
Total	404.87 (358.85)	171.34 (151.86)	0.255	1,587.73	671.92	—
Methotrexate	95.06 (105.71)	40.23 (44.74)	0.254	374.25	158.39	1
Leflunomide	749.67 (228.42)	317.25 (96.96)	0.282	2,658.40	1,125.00	4
Sulfasalazine	251.58 (137.47)	106.47 (58.17)	0.176	1,429.43	604.94	3
Ciclosporin	230.73 (107.09)	97.64 (45.32)	0.195	1,183.23	500.72	2
*p*-value	<0.001^a^	<0.001^a^	—	—	—	—

BRL: brazilian real; PPP: purchasing power parity.

a < 0.001 for all comparisons, except for ciclosporin versus sulfasalazine.

Despite being the drug with the best medication persistence, leflunomide was the drug with the highest cost. In this sense, leflunomide had the highest cost per responding patient. Methotrexate, on the other hand, had the lowest drug cost and the lowest cost per responder being considered the most efficient drug.

## Discussion

This is the first national study in Brazil comparing multiple csDMARDs for psoriatic arthritis. These are significant findings, indicating that methotrexate has the best cost-benefit ratio, while leflunomide has the best treatment persistence but the highest cost of all drugs assessed.

Some studies have observed the performances of csDMARDs in the treatment of psoriatic arthritis (Farr et al., 1988; Gupta, 1989; Farr et al., 1990; Combe et al., 1996; Fraser, 2005; Malesci et al., 2007; Ricci et al., 2011; Behrens et al., 2013; Nikiphorou et al., 2014; Landi et al., 2018; Ribeiro da Silva et al., 2019; Jacobs et al., 2020; Maksabedian Hernandez et al., 2020), among which five allow the comparison of drugs (Malesci et al., 2007; Landi et al., 2018; Ribeiro da Silva et al., 2019; Jacobs et al., 2020; Maksabedian Hernandez et al., 2020), with increasing the relevance of these findings. In addition, one clinical trial has been conducted to assess methotrexate for PsA (Mulder et al., 2020). As a result, the findings are important to better understand the reality of treatment with these drugs in a real-world setting. It was observed that the patients had a mean age of 51.11 years, with the highest proportion (31.4%) in the group with an age range between 46 and 55 years, which corroborates data from the literature showing that the peak incidence of PsA occurs between the fourth and fifth decades of life (Liu, 2014). In a multicenter study in Italy involving 37 rheumatology centers, the mean age found was 49 years (Cervini et al., 2011). In the United States, an epidemiological study identified that disease onset occurs on average at 46.4 years (Karmacharya et al., 2021).

Considering that the use of conventional synthetic disease course modifying drugs are the first line of treatment for psoriatic arthritis, one can infer that the average age of diagnosis of psoriatic arthritis in Brazil is around 50 years old. When compared to data from the United States, which indicate an onset of the disease at 46.4 years of age, possible difficulty in diagnosing the disease in Brazil can be investigated. Clinical guidelines indicate that delay in diagnosis is a major challenge that needs to be addressed, as it negatively impacts treatment outcomes. Thus, strategies to promote early referral and decrease the delay in diagnosis and treatment of inflammatory arthritis are needed (Gossec et al., 2015; Haroon et al., 2015).

This is a problem that has been faced in Brazil and one of the challenges encountered is represented by the concentration of rheumatology physicians in large cities and the low availability of rheumatologists in the public health system (da Silva et al., 2019a; da Silva et al., 2019b).

The present study showed a slight predominance of females (60.1%), which is common in other studies conducted in Brazil. However, in studies with large databases, a similar distribution of the disease between genders is usually observed (da Silva et al., 2019b).

Among the drugs evaluated in the cohort, methotrexate was the most used among patients, followed by leflunomide, ciclosporin, and sulfasalazine. This finding corroborates the clinical protocols for the treatment of PsA, where methotrexate is recommended as the first choice for the treatment of the disease. Methotrexate is recommended for the treatment of peripheral joint and skin involvement in PsA, preferably at a dose greater than 15 mg per week subcutaneously, due to the adverse events seen with the oral route. If methotrexate is not available, ciclosporin, leflunomide or sulfasalazine should be used in patients with peripheral arthritis (Coates and Helliwell, 2015; Gossec et al., 2015; Singh et al., 2018; Carneiro et al., 2021).

According to Kane and collaborators, methotrexate was the most prescribed csDMARD in an American hospital. Despite clinical improvement with csDMARD use, 47% of patients had radiological damage at a median interval of 2 years (Kane, 2003). Leflunomide has been evaluated in a few observational studies and has shown benefits in improving peripheral and skin outcomes, with concomitant use with methotrexate leading to a greater likelihood of achieving a 50% improvement in the Psoriasis Area Surface Index (PASI50). Additionally, benefits were observed in the control of pain, fatigue, and dactylitis (Behrens et al., 2013).

Methotrexate is one of the most widely used cDMARDs worldwide for the treatment of PsA, although few clinical trials have evaluated its efficacy, and clinical evidence is still limited (Fraser, 2005; Coates and Helliwell, 2015).

Old clinical trials, with small sample size, indicated that the use of sulfasalazine in the treatment of PsA is safe but had a limited efficacy (Combe et al., 1996; Farr et al., 1990; Farr et al., 1988). Limited clinical evidence is available for ciclosporin in the treatment of PsA, which indicates possible benefits from its use (Gupta, 1989). In combination with methotrexate, ciclosporin appears to control inflammation but not pain and quality of life for patients (Fraser, 2005).

Medication persistence at 6 months was 58.9% for leflunomide, 51.6% for methotrexate, 44.8% for sulfasalazine, and 42.7% for ciclosporin. There was a significant decrease in medication persistence after 120 days of the start of therapy, which was due to the first renewal of treatment in the SUS occurring during this time (treatment renewal occurs every 3 months). Following discontinuation, part of the patients switched the therapy, mainly to a biological drug (da Silva et al., 2019a; da Silva et al., 2019b). At 12 months, medication persistence reduced to 28.2% for leflunomide, 25.2% for methotrexate, 19.5% for ciclosporin, and 17.6% for sulfasalazine. Therefore, differences in medication persistence were minimal for leflunomide and methotrexate.

There are no clinical trials that directly compare csDMARD for the treatment of PsA (Kang and Kavanaugh, 2015). Additionally, few observational studies have evaluated more than one csDMARD for PsA, with medication persistence the most common outcome reported (Ribeiro da Silva et al., 2019; Jacobs et al., 2020). In a retrospective cohort study with 187 adult PsA patients in the Netherlands, patients using first-line methotrexate presented higher medication persistence than ones using sulfasalazine (log-rank < 0.05). At 1 year of treatment, patients on methotrexate had a retention rate of approximately 70%, while patients on sulfasalazine had 50%. The main reasons for csDMARD retention failure in PsA are treatment inefficacy (52%) and side effects (28%) (Jacobs et al., 2020).

In an Argentine cohort study, 87 adult PsA patients completed the follow-up. According to the findings, methotrexate was the most commonly used csDMARD, followed by leflunomide. Methotrexate had a higher cumulative survival rate than leflunomide and was aided by concomitant steroid therapy, whereas leflunomide had a higher survival rate in elderly patients (Landi et al., 2018).

In a retrospective study with 63 patients using methotrexate and leflunomide in Brazil, no difference was observed in the medication persistence. At 12 months, 37.7% of patients on leflunomide and 34.0% on methotrexate remained on treatment (Ribeiro da Silva et al., 2019).

Overall, medication persistence with csDMARDs is lower than biological drugs in Brazil (da Silva et al., 2019b) and other countries (Murage et al., 2018; Murray et al., 2021). According to Murage and collaborators, medication persistence for TNF inhibitors can vary from 50 to 75% at 12 months, depending on biologic drug in use (Murage et al., 2018). Murray and collaborators found an overall medication persistence of 59% at 12 months for biological therapy in psoriatic arthritis (Murray et al., 2021).

In this sense, there is a rapid shift from synthetic to biological therapy, and the reasons for this must be investigated, owing primarily to the failure of synthetic treatment and the higher cost of biological therapies (da Silva et al., 2019b; Maksabedian Hernandez et al., 2020). Thus, observational studies in Brazil and other countries evaluating the effectiveness and safety of these drugs for psoriatic arthritis could be recommended.

In this study, patients who were treated with leflunomide and methotrexate were the most persistent, while individuals taking ciclosporin and sulfasalazine showed a higher rate of discontinuation in the treatment of PsA. Methotrexate and leflunomide are usually the csDMARD investigated in observational studies for PsA, and the results are comparable between them (Landi et al., 2018; Ribeiro da Silva et al., 2019). Sulfasalazine appeared only in one study versus methotrexate, with a worse result of persistence (Jacobs et al., 2020).

Methotrexate had a medication persistence of 51.6% at 6 months and 25.4% for 12 months. These findings differ from a study conducted in Italy that found 80 and 69% persistence for 6 and 12 months, respectively (Ricci et al., 2011). Another American study brings similar results to those found in this research, where 34.1 and 25.2% of patients remained in treatment with methotrexate and sulfasalazine respectively after 1 year of follow-up (Maksabedian Hernandez et al., 2020).

In summary, differences in medication persistence have been observed when comparing studies (Landi et al., 2018; Ribeiro da Silva et al., 2019; Jacobs et al., 2020). This can be explained by differences concerning organization and access to health services, arising from regional inequities and methodological differences between studies.

This is corroborated by the lower medication persistence observed in patients living in the North and Northeast regions of the country, since these regions have worse social and economic indicators, in contrast to the South and Southeast regions, with better economic and social indicators. In Brazil, access to health services is strongly influenced by the supply of supplementary health services, people’s social status, and where they live (Kang and Kavanaugh, 2015). On the other hand, access improvements have already been observed in the North and Northeast regions in recent years (Cambota and Rocha, 2015; Albuquerque et al., 2017).

Younger individuals had a higher discontinuation rate. This finding is similar to an Argentine cohort, which found that patients older than 50 years treated with leflunomide had a higher persistence to treatment (Landi et al., 2018). This effect was also observed for methotrexate, but the patients treated with this drug were on steroids (Ribeiro da Silva et al., 2019).

In terms of drug costs, leflunomide showed the highest cost, followed by sulfasalazine, ciclosporin, and methotrexate. Methotrexate was the drug being the lowest cost per response. Despite leflunomide demonstrating superior medication persistence, its higher cost is a disadvantage when compared to other csDMARD. In this regard, lowering the cost of leflunomide may improve its efficiency for PsA (Gupta, 1989). In addition, drug costs for csDMARDs are very less than biological drugs (Gupta, 1989; Ricci et al., 2011).

This study has advantages and limitations. As for advantages, it is noteworthy that this is the first study with a large sample size to evaluate csDMARD for the treatment of psoriatic arthritis. This is of particular importance given the scarcity of studies evaluating these drugs. Additionally, the use of Unified Health System databases can contribute to the generation of useful knowledge to reassess and support decision-making in health. In this sense, it appears that Brazil has a large amount of data that has been organized to carry out pharmacoepidemiological studies (Guerra Junior et al., 2018; Leal et al., 2022).

As for disadvantages, we mention the impossibility of identifying the causes of treatment discontinuation, such as ineffectiveness, side effects, among others. In addition, it was not possible to stratify patients using oral and subcutaneous methotrexate. Furthermore, this database lacks clinical data on disease activity, which was one of the study’s limitations. At last, the data were paired with the identification of the patient’s line of care until 2015, which precluded analysis of a more recent period.

## Conclusion

The current study adds to the understanding above the use of csDMARDS for the treatment of PsA. Methotrexate and leflunomide were the most used csDMARDs. Methotrexate had the best cost per response ratio, owing to its lower cost and a slightly lower proportion of persistent patients when compared to leflunomide. Leflunomide had the highest medication persistence, but it was also the most expensive drug. The rate of treatment discontinuation was relatively high for all drugs. As a result, it is recognized that there is a need for the development of actions aimed at improving outcomes related to psoriatic arthritis treatment to contribute to better pharmacotherapy for these patients.

## Data Availability

The data that support the findings of this study are available from the corresponding author upon reasonable request.
